# Can personal qualities of medical students predict in-course examination success and professional behaviour? An exploratory prospective cohort study

**DOI:** 10.1186/1472-6920-12-69

**Published:** 2012-08-08

**Authors:** Jane Adam, Miles Bore, Jean McKendree, Don Munro, David Powis

**Affiliations:** 1 Hull York Medical School, University of York, York YO10 5DD, UK; 2 School of Psychology, University of Newcastle, Callaghan, NSW, 2308, Australia

## Abstract

**Background:**

Over two-thirds of UK medical schools are augmenting their selection procedures for medical students by using the United Kingdom Clinical Aptitude Test (UKCAT), which employs tests of cognitive and non-cognitive personal qualities, but clear evidence of the tests’ predictive validity is lacking. This study explores whether academic performance and professional behaviours that are important in a health professional context can be predicted by these measures, when taken before or very early in the medical course.

**Methods:**

This prospective cohort study follows the progress of the entire student cohort who entered Hull York Medical School in September 2007, having taken the UKCAT cognitive tests in 2006 and the non-cognitive tests a year later. This paper reports on the students’ first and second academic years of study. The main outcome measures were regular, repeated tutor assessment of individual students’ interpersonal skills and professional behaviour, and annual examination performance in the three domains of recall and application of knowledge, evaluation of data, and communication and practical clinical skills. The relationships between non-cognitive test scores, cognitive test scores, tutor assessments and examination results were explored using the Pearson product–moment correlations for each group of data; the data for students obtaining the top and bottom 20% of the summative examination results were compared using Analysis of Variance.

**Results:**

Personal qualities measured by non-cognitive tests showed a number of statistically significant relationships with ratings of behaviour made by tutors, with performance in each year’s objective structured clinical examinations (OSCEs), and with themed written summative examination marks in each year. Cognitive ability scores were also significantly related to each year’s examination results, but seldom to professional behaviours. The top 20% of examination achievers could be differentiated from the bottom 20% on both non-cognitive and cognitive measures.

**Conclusions:**

This study shows numerous significant relationships between both cognitive and non-cognitive test scores, academic examination scores and indicators of professional behaviours in medical students. This suggests that measurement of non-cognitive personal qualities in applicants to medical school could make a useful contribution to selection and admission decisions. Further research is required in larger representative groups, and with more refined predictor measures and behavioural assessment methods, to establish beyond doubt the incremental validity of such measures over conventional cognitive assessments.

## Background

In recent decades it has been generally recognised that, in addition to academic ability, doctors’ professional competence depends on a range of desirable personal qualities (for example [1]). These have been recently summarised [2] as teamwork and professional skills, duty and responsibility, professionalism and values, communication and interpersonal skills, and trustworthiness and ethical behaviour. It is also now recognised that medical schools have a duty to society to select as students those individuals most likely to graduate as doctors with such attributes [3].

To achieve this, some universities have been investigating the use of tests of cognitive and non-cognitive personal qualities to augment their selection procedures for medical students. In the UK 26 of the 32 medical schools have been exploring the use of the United Kingdom Clinical Aptitude Test (UKCAT) [4]. The use of such tests is controversial and clear evidence of their predictive validity continues to be lacking [5-7] because studies combining the administration of these tests with observational measures of student behaviour (or professional behaviour after graduation) have not been carried out. In addition the literature lacks reports of whether the non-cognitive characteristics desired in a good doctor have any bearing on students’ examination results, particularly in performance-based exams. Our aim was therefore to establish whether any of a range of tests of cognitive abilities and non-cognitive personal qualities [8-11] that have been developed for medical student selection can predict professionally appropriate behaviours in medical students, in addition to their normal examination results.

At Hull York Medical School (HYMS), students’ marks from their annual summative theme-based examinations can be broadly attributed to one of three domains: recall and application of knowledge, evaluation of data, and communication and practical skills, the latter including ratings from objective structured clinical examinations (OSCEs). HYMS tutors undertake regular formative assessments of students’ professional behaviours, understanding and performance from the first year of the course onwards, in order to get closer to the ideal of a comprehensive assessment. Using a set of rating scales, these assessments record behaviours acknowledged as important by medical educators. This includes behaviours reflecting conscientiousness (such as punctuality and appropriate dress), interactions with tutors and fellow students, involvement in the group learning experience, and appropriate clinical behaviours. The tutor ratings provide a novel, but appropriate, set of outcome variables against which to examine the predictive validity of selection tests.

The range of information available from the HYMS assessments thus allowed exploration of whether the UKCAT cognitive and pilot non-cognitive tests of personal qualities can predict either examination performance or the surrogate measures of professional behaviours as observed by the tutors.

## Methods

**Figure 1:**
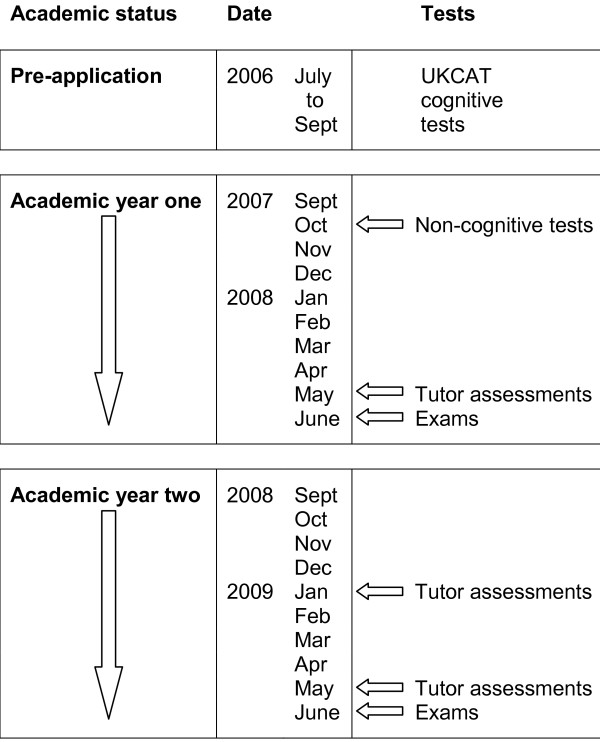
**Schedule of testing from pre-application to end of academic year two. **Timing of cognitive and non-cognitive qualities tests, tutor assessments and end of year examinations.

This paper reports the first two academic years’ progress of the 146 students who, in September 2007, commenced year 1 of the five-year medical course at Hull York Medical School (HYMS). All were invited to participate in the study, which had ethics approval from HYMS’ Medical Education Ethics Committee (study ref 0701). Data were collected from the following measures. Figure 1 shows when each set of measurements was undertaken.

1. The UKCAT test, which all students were required to have taken in 2006 before applying to medical school, provided four cognitive skills subtest scores: *verbal reasoning (VR), numerical reasoning (NR), abstract reasoning (AR)* and *decision analysis (DA)*[4]. HYMS did not preselect students on the basis of any minimum or specified range of UKCAT performance. UKCAT results were available for 131 students; all but two of the remaining students had applied a year earlier, before the UKCAT was introduced.

2. The non-cognitive personal qualities assessments were three paper-based tests delivered under examination conditions at the University of Hull and the University of York in October 2007. The tests, which have also been part of the UKCAT since 2007, were:

a) The Interpersonal Traits Questionnaire (ITQ), which measures *narcissism, aloofness, confidence* (in dealing with people) and *empathy* and produces a summary score for *INVOLVEMENT* (versus detachment) in which *confidence* and *empathy* are positive, *narcissism* and *aloofness* negative [8,11].

b) The Interpersonal Values Questionnaire (IVQ), which measures the extent to which the respondent favours i*ndividual freedoms (*versus *societal rules)* as a basis for making moral decisions [9,11].

c) The Self-Appraisal Inventory (SAI) [11], which measures the domains of (mental) *RESILIENCE* (comprising scales measuring *anxiety, moodiness, neuroticism* and *irrational thinking*) and *SELF-CONTROL (*versus risk taking tendency) using the scales of *restraint*, *conscientiousness*, *permissiveness* and *anti-social tendencies*. SAI also contains a Lie scale.

3. Tutor assessment (TA) data are collected routinely about all students, from each problem-based learning (PBL) tutor. Groups of 8 students meet with the same tutor twice a week for a 1.5 h problem-based learning (PBL) tutorial throughout year 1, and again (in different groups with different tutors) throughout year 2. These tutors, all clinicians, are also the personal mentor for their students, and consider each individual’s assessment data in formative one-to-one interviews between tutor and student. These data comprised:

a. Assessment of a set of specified behavioural items, adapted from the *Peer assessment of professional behaviours form* created by Gary Butler, University of Wollongong, and used with permission. The items were suggested by the requirements for medical school curricula [3], taking into consideration the ability of tutors to observe student behaviour. The assessments were undertaken by their PBL tutor for each student once in year 1 (in May 2008) and twice in year 2 (January and May 2009). The assessment form was being developed over this period; it was piloted in May 2008, revised in January 2009 and finalised in May 2009, so there was slight variation in the items on each occasion, with the tutors assessing 17 behaviours in May 2008 and May 2009, but only 14 behaviours in January 2009. See Additional file 1 for an example of each form. The final three items (18 to 20) on the May 2009 form are excluded from the analysis because these items relate to a different context (clinical placements) and were assessed by other tutors whom the students met irregularly.

b. In addition, as part of this research, the PBL tutors were asked to make an overall assessment of each student in May each year, by rating them as either ‘problematic’, ‘average’ or ‘particularly promising’.

4. The marks from end of academic year summative examinations, held in June 2008 and June 2009, which are allocated to one of three HYMS themes: Theme A (*Life sciences* and *Clinical sciences*), Theme B (*Clinical techniques and skills* and *Person-centred care*) and Theme C (*Evidence-based decision-making, Population health and medicine* and *Managing resources for quality and efficiency*). Theme A tests largely knowledge recall, with some interpretation. Theme B tests interpersonal understanding and communication and practical skills by a written paper (30% of marks) and an OSCE (70%). The OSCE included equal numbers of five minute stations covering practical skills and communication skills; for a detailed list of the stations, see Additional file 2. Theme C tests not only knowledge, but also analytic and numerical evaluation skills.

Tutor assessments and examination data were available for the whole cohort of 146 students. 143 agreed to take part in the non-cognitive study but only 137 completed all three non-cognitive tests, of whom 122 had also completed the UKCAT cognitive tests (see Additional file 3).

The data were entered into SPSS for analysis and screened for abnormal distributions. As this was an exploratory rather than a hypothesis driven study, structured methods such as regression analysis and adjustment of significance levels for repeated comparisons were eschewed in favour of describing basic relationships between variables, and the effects of collinearity were not taken into account. The boundary provided by statistical significance was taken as a guide to which relationships are reported as important.

Pearson product–moment correlations were computed within and between the groups of measures given above, and significant results (p < .05, 2-tailed test) tabulated. Although only four rating categories (Unsatisfactory, Borderline, Satisfactory and Excellent) were available to tutors, these scales were assumed to approximate interval measurements and to be suitable for parametric statistical tests. Correlations involving variables with some missing data were automatically adjusted on a case-by-case basis. To assess relationships with high and low medical school performance, scores on non-cognitive tests, tutor assessment (TA) items, overall tutor rating and OSCEs were compared for students obtaining the top and bottom 20% of the summative examination results, using one-way Analysis of Variance to produce an F value.

## Results

**Figure 2:**
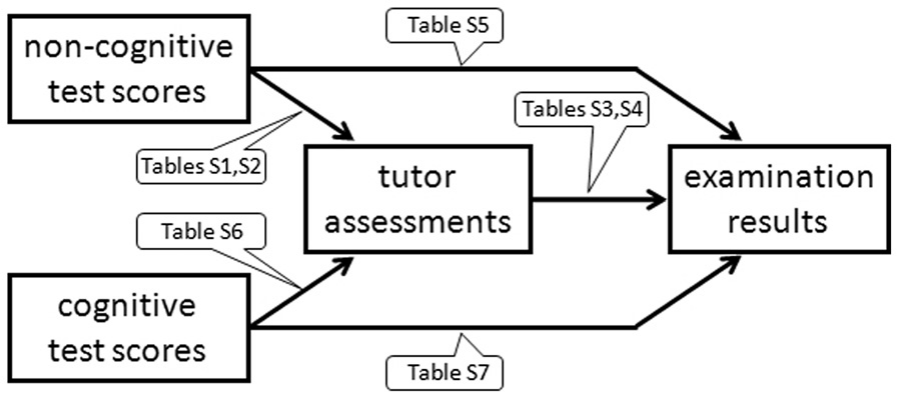
Relationships explored in text and tables.

This study reports students’ progress up to the end of year 2, including performance in the year 1 and year 2 summative examinations. Figure 2 summarises the relationships explored. The significant results (p < .05, 2-tailed test) among the Pearson product–moment correlations for each group of data are shown in the following tables, along with significant F values obtained by comparing students obtaining the top and bottom 20% of the summative examination results using Analysis of Variance. In the interests of clarity, variables with non-significant results (personality and tutor ratings) are omitted and revealed as blank cells in the tables.

### Structure of the tutor assessment scales

The structure of the tutor assessment scales was analysed for each session using reliability analysis. The internal consistency of the scales was high in all cases, the Cronbach alpha coefficients being 0.91 for May 2008 (17 items), 0.87 for January 2009 (14 items), and 0.90 for May 2009 (20 items), indicating that there was a strong tendency for the tutors to give similar ratings to individual students for all items in the scales.

### Prediction of tutor assessments by non-cognitive tests

The significant relationships found between non-cognitive characteristics and tutor assessments in year 1 and year 2 are shown in Additional file 4: Table S1 and Additional file 5: Table S2 respectively.

From the SAI test, high *neuroticism* (p < .05), *moodiness* (p < .05), *irrational thinking* (p < .05 to p < .001)*,* and low *conscientiousness* (p < .05) predicted lower ratings by tutors for some individuals’ behaviours in year 1. These traits combine into the SAI factor *RESILIENCE,* which itself predicts some positive behaviours of individuals in the first year PBL groups (p < .05). In year 2, the SAI factor *SELF-CONTROL* (incorporating + r*estraint,-permissiveness, -antisocial behaviour*) (p < .05 to p < .001) and *conscientiousness* (p < .05 to p < .01) predict some positive behaviours of individuals, while the individual traits *permissiveness* and *anti-social behaviour* both correlate negatively (p < .05) with some year 2 behaviours.

All of the individual measures of the ITQ (*empathy, confidence, narcissism* and *aloofness*) correlate with some tutor assessed behaviours of individuals in year 1 and in year 2 (p < .05 to p <0.001) and, in general, the correlations are in the direction that is expected intuitively, for example *narcissism* and *aloofness* are negatively correlated with positive behaviours. The combined ITQ factor *INVOLVEMENT* (*empathy, confidence,* low *narcissism,* low *aloofness*) correlates positively with the overall tutor assessment in year 1 (p <0.05) but not in year 2. In year 2 only, *social responsibility* (IVQ test) predicts appropriate self-management (rather than group-orientated) behaviours (p < .05 to p < .01).

### Prediction of examination results by tutor assessments

The tutor assessments focus on a range of desirable professional behaviours. Many, but not all, items predict aspects of examination performance in both year 1 and year 2, as shown in Additional file 6: Table S3 and Additional file 7: Table S4. For both years, those tutor assessment items that related to the students’ participation in the functioning of the group (for example, *contribution of work to the group*, and *contribution to the positive atmosphere in the group*) best predicted overall examination performance (including OSCE skills), and best predicted the differences between the best and worst performing students in their examinations (top 20% vs bottom 20% of marks range). Somewhat surprisingly, there are more significant relationships with Theme A scores (recall of scientific knowledge) than with Theme B (clinical techniques and skills, including OSCEs).

### Prediction of examination outcomes by non-cognitive subtest scores

The non-cognitive traits that predicted examination outcomes are shown in Additional file 8: Table S5. Greater *narcissism* and *aloofness* (from ITQ), and *irrational thinking* (from SAI), predicted poorer overall examination scores (p < .05), and poorer performance in Theme A, Theme B overall and communication skills OSCEs. *Conscientiousness* (from SAI), *confidence* (from ITQ) and the *INVOLVEMENT* factor score all predicted better performance in the practical skills OSCEs (p <0.05 to p <0.001). *Narcissism* (p <0.05), *aloofness* (p < .001) and the *INVOLVEMENT* factor score (p < .05) each differentiated between the best and the worst performing students in the year 1 examinations. Non-cognitive test scores did not predict examination performance in Theme C.

### Prediction of tutor assessments by UKCAT cognitive test scores

UKCAT cognitive tests scores did not predict many of the behaviours subsequently rated by tutors, as shown in Additional file 9: Table S6. In year 1, no behavioural items in the year 1 tutor assessments (May 2008) correlated with the overall UKCAT cognitive ability test score, and only one (negative) correlation was found with a single subtest score. Although the year 2 tutors undertook assessments twice, only eight significant correlations between the behavioural measures and cognitive test scores were found. The *abstract reasoning* test score predicted four behaviours in the May 2009 assessment by tutors*.* The *UKCAT cognitive test overall score* correlated with only one rated behaviour from the May 2009 tutor assessment and no behaviour from the May 2008 and January 2009 tutor assessments.

### Prediction of examination outcomes by UKCAT cognitive test scores

The *UKCAT cognitive test overall score* and the *decision analysis* subtest score were significant predictors for both year 1 and year 2 overall exam scores (p < .001), and performance in Theme A (which tests knowledge acquisition) (p < .05 to p < .01), as shown in Additional file 10: Table S7. Both were also strong predictors in each year of the best versus worst examination performers. Performance in Theme C (which requires acquired knowledge and the application of numerical and analytical skills) was the result best predicted each year by both the overall cognitive ability score (p < .001) and by each subtest score. Cognitive test scores did not predict performance in the communication skills components of the clinical examinations (OSCEs) in either year; physical examination skills correlated positively only with *decision analysis* and only in year 1. Overall performance in theme B (patient centred-care, communication and interpersonal understanding) was predicted, but only in year 2, by three of the four cognitive test scores.

### Reliability of tutor assessments

Additional file 11: Table S8 shows the re-test reliabilities of those behaviours assessed at least twice, by different tutors (year 1 tutor: May 2008; year 2 tutor: January 2009, May 2009). The reliabilities of the overall tutor ratings are high (p < .001), as are the reliabilities of the items that relate to group characteristics, such as *integrates into group, demonstrates appropriate attitudes, takes responsibility for group learning, contributes work for the group,* and *willing to learn from others* (p < .01 to p < .001).

## Discussion

This study rests on the co-incidence of three fortunate sets of circumstances. First, it was possible to follow an entire medical school entry cohort of students, the majority of whom had taken the UKCAT cognitive tests but the scores had not been used in the students’ selection (so there was no restriction of range within the study population) and then for research purposes these students took the same non-cognitive tests that were included within the subsequent years’ UKCAT. Secondly, the tutor assessments provide perhaps the closest approximation to all-round evaluation of individual professional performance during the early years of a medical course. Thirdly, the HYMS theme-based examination system requires allocation of examination marks into the three separate themes that depend mainly on scientific knowledge (Theme A), on clinical information gathering and inter-personal skills (Theme B) and on application of statistical and analytical skills to acquired knowledge (Theme C), thus allowing different aspects of students’ performance to be distinguished.

### Principal findings

This study has found numerous significant relationships between students’ prior cognitive and non-cognitive test measures (all of which became components of the UKCAT), subsequent tutor assessments of individual and group-related behaviours, and academic and clinical examination results undertaken over the first two years of a medical school course.

The individual predictive ability of any of the measures (of cognitive skills, non-cognitive traits and of behaviour) appears weak, the majority of the statistically significant correlations being in the range 0.16 to 0.24 and only a few exceeded 0.30 (accounting for only 9% of the variance). However, in the field of organisational psychology it is well recognised that even quite weak correlations are useful predictors of workplace outcomes, especially if the ratio of applicants to selectees is large [12], as with applications to medical school. Such measures may therefore usefully predict medical student performance and later professional conduct. This position is supported by a recent meta-analysis of the predictive value of ability and personality test scores, which concluded that such scores are more successful in predicting educational, work and life outcomes than is often admitted by critics; in particular “In medical education, personality characteristics gain importance for later academic performance when applied practice (such as performance in practicums and clerkships) increasingly plays a part” [13].

A further possible criticism of the findings is that there are relatively few significant relationships, as revealed by the number of blank cells in Additional file 4: Table S1 and Additional file 5: Table S2 particularly. However, of the 380 possible relationships that could have been included in these tables (19 personality scales by 20 tutor ratings), 51 (or 13.4%) were significant at the 5% level or better; this becomes 18.2% of 280, if only the 14 personality scales actually appearing in the tables are considered. When compared with the chance proportion of 5% it is clear that the overall pattern of results is better than chance. Additional analyses were conducted to adduce further evidence for the significance of the overall pattern, including multiple regression to calculate the proportion of common variance shared by the personality scales and the tutor ratings, and application of a recently published resampling technique [14] for calculating the probability of relationships between personality and behaviour. However, neither provided stronger support than the simple number of correlations, so the details are not presented here.

In summary, although the statistically significant coefficients in the matrices could all be chance findings this seems unlikely because all of the significant results (and most of the non-significant ones, which are not shown) are in the direction that the particular personality traits and cognitive skills would be expected to affect the behavioural and examination outcomes.

### The findings in more detail

Overall, it appears that the non-cognitive tests do predict normally unrecorded aspects of medical students’ performance. Greater *narcissism*, *aloofness* and *irrational thinking* predicted lower tutor ratings for group-related behaviours, and poorer overall examination performance including in the most knowledge-based Theme A, as well as in Theme B (person-centred care), though not in Theme C. Good teamwork skills therefore appear to be an important student attribute. The finding that *RESILIENCE* predicted good group functioning in year 1, while c*onscientiousness* and *SELF-CONTROL* predict this in year 2, may reflect students’ increasing ease and familiarity with medical school. The early years of the HYMS course involves not only problem-based learning sessions but also a structured weekly programme of lectures, laboratory-based practical classes and tutored clinical experience, so the importance of group functioning to the examination outcomes cannot be explained by exclusive reliance on problem-based group learning.

While the UKCAT cognitive tests scores did not predict many of the behaviours subsequently rated by tutors, they did predict both year 1 and year 2 overall examination scores, and performance in Theme A and Theme C. Notably, but not unexpectedly, cognitive test scores did not predict performance in the communication skills or physical examination components of the clinical examinations (OSCEs) in either year, although they did predict overall Theme B scores at the end of year 2. This may reflect increasing emphasis in year 2 on clinical reasoning skills within this theme.

### Problems with predicting traditional medical school results

Prediction of any outcome depends on selection and accurate measurement of both predictor (be that a cognitive skill or a personality trait) and an appropriate outcome (for example, academic marks, skills, or professional behaviours). In order to examine whether any of the cognitive and pilot non-cognitive components of the UKCAT are valid predictors it is necessary to have appropriate measures of the behaviours that the tests are expected to predict. Paradoxically, in-course examinations do not usually assess outcomes that are related to the qualities that non-cognitive selection tests are designed to measure. In general, medical schools have concentrated on traditional examinations (in part so that licensing requirements can be fulfilled unambiguously) that test recall of factual material and reasoning, which depends on memory and cognitive skills. Non-cognitive skills, such as communication and doctor-patient relationships, are often tested informally within medical training, and the results recorded in pass/fail format, usually with only a small number of failures (as with many academic medical examinations). Thus a problem for statistical comparisons of traditional medical school results is that pass/fail clinical outcomes are categorical and typically severely skewed while the predictor test results are normally distributed on a continuous scale. The present study benefits from finer gradation in both selection tests and the outcomes measured, and from better matching between predictor and criterion variables.

### Other studies

Cognitive tests would be expected to predict success in examinations of knowledge recall. This has been shown in one recent study [15] about medical students, but not in others [5-7]. No reported studies have been found that tested the hypothesis that non-cognitive characteristics desired in a good doctor have any bearing on students’ examination performance, although a Conscientiousness Index [16] has been proposed as a measure of medical students' professionalism based on a variety of routinely made behavioural observations that can be recorded in a systematic and reliable way. The present study is the first to demonstrate that measurement of a range of different personal qualities can predict different aspects of medical students’ performance. The broad range of suitable measures of non-cognitive skills and professional behaviours used here provides possible tools for future research.

### Relevance of this study: implications for clinicians and policymakers

All health professionals require good communication skills and the ability to work effectively as part of a team. Doctors have additional roles, not only in education and in research but also, principally, as decision-makers and leaders of teams working in situations of clinical complexity and uncertainty. All doctors therefore should be committed to reflective practice, monitoring their own contribution and working continually to improve their own and their team’s performance [17]. Selection tests need to encompass more than purely cognitive skills, and test for the other abilities that underpin the whole range of the doctor’s professional tasks.

In the present study, non-cognitive tests were found to predict behaviours likely to be important when working as a doctor, such as functioning well with others in groups, acknowledging weaknesses and accepting feedback (which underpins the ability to learn and change through experience), and identified other traits, such as *narcissism*, *aloofness* and *irrational thinking*, that are likely to diminish a doctor’s ability to fulfil these roles. Improvement in the predictive power of such non-cognitive tests depends not only on finding better tests, but on devising and using better measures of critical behaviours in medical school and professional practice.

### Unanswered questions and future research

The findings raise three further questions with implications for medical education. First, in the selection of future medical students, should the predictive ability of cognitive and non-cognitive qualities be explored more fully, particularly in relation to the difficult judgments and high level of inter-personal skills required of a doctor? Secondly, should medical schools be making use of more standardised and repeated behavioural observations undertaken by tutors throughout the students’ training, in addition to current measures of clinical competence (such as mini-clinical evaluation exercise [18]) used in formative and summative assessments of medical students? Thirdly, are medical schools failing to assess all the appropriate outcomes from their courses by continuing to rely too heavily on formal examinations based mainly on recall of acquired knowledge and some reasoning? The latter approach has produced many doctors who are intellectually and academically prepared for their careers, but personal failings, such as poor communication skills, lack of empathy and concern for patients, motivation, and mental health issues, tend to impinge on their work effectiveness. Such failings are typically detected too late, when brought to light by examination failure or the need for disciplinary action. If it is accepted that doctors require more than just academic knowledge and technical skills then it makes sense to look for additional qualities at the outset and select those applicants who already have these qualities or seem capable of developing them.

## Conclusions

The findings of this study as a whole reveal a pattern of relationships between cognitive and non-cognitive factors and medical school performance consistent with intuitive and theoretical expectations. Our study suggests that measurement of non-cognitive personal qualities in applicants could improve the selection of medical students, especially in regard to performance in the inter-personal skills and professional behaviours needed by doctors. However, further research is needed into the best non-cognitive measures for the prediction of various target skills and behaviours, and into the degree to which such measures can improve on the predictive validity of existing cognitive selection measures. If such research confirms which personal qualities are most important, then it may indeed be possible to succeed in the aim of producing doctors that meet better the expectations of twenty-first century patients.

## Competing interests

JA was an unpaid member of the UKCAT executive board from 2005 to 2010. JA and JMcK jointly received £2000 from The University of Newcastle Research Associates Ltd (TUNRA), now known as Newcastle Innovation Ltd., on behalf of HYMS to pay for the costs of running of the tests and data entry.

MB, DM and DP have support from The University of Newcastle Research Associates (TUNRA), now known as Newcastle Innovation Ltd., for the submitted work, and have in the last three years had commercial relationships with Pearson VUE, a contractor for UKCAT.

The authors’ spouses, partners, or children have no financial relationships that may be relevant to the submitted work.

## Authors’ contributions

JA and DP had the initial idea. JA and JMcK enrolled and tracked the cohort, administered the non-cognitive questionnaires and collated the HYMS exam performance data. JMcK developed the tutor rating questionnaires. DP, MB and DM have developed and extensively tested the non-cognitive questionnaires over 14 years. DM performed the statistical analyses. JA and DP wrote the initial draft of the paper with subsequent contributions from all authors. All authors had full access to all of the data (including statistical reports and tables) in the study and take responsibility for the integrity of the data and the accuracy of the data analysis. All authors amended and approved the article for submission.

## Authors’ information

JA was Associate Dean for Admissions at Hull York Medical School from 2003 to 2011, and has a continuing research interest in methods for selecting medical students.

MB is a senior lecturer and researcher in the School of Psychology at the University of Newcastle, Australia with particular interests in the areas of personality, psychometrics, moral orientation.

JMcK is Associate Dean for Assessment and Senior Lecturer in Medical Education at Hull York Medical School. She is a cognitive psychologist whose research includes applying cognitive science principles to educational areas such as educational technology, organisational redesign, the role of discussion in learning, graphical representations for reasoning in and across disciplines, and effectiveness of assessments.

DM is an Associate Professor in the School of Psychology at the University of Newcastle, Australia, and a member of the PQA research group. His main interests are in the areas of personality, psychometrics and selection test construction.

DP is Conjoint Professor at the University of Newcastle, Australia. He has been a university teacher of, and researcher in, physiology and medical education since 1972. He has developed a professional interest in the area of medical student selection with the aim of establishing fair principles and appropriate procedures for selecting students for health professional courses. Since 1997 he has worked with Miles Bore and Don Munro to develop and evaluate the Personal Qualities Assessment (http://www.pqa.net.au) as an instrument for this strategic aim.

## Pre-publication history

The pre-publication history for this paper can be accessed here:

http://www.biomedcentral.com/1472-6920/12/69/prepub

## Supplementary Material

Additional file 1 **Examples of Tutor assessment forms. **(i)Tutor assessment form, May 2008. (ii)Tutor assessment form, January 2009. (iii)Tutor assessment form, May 2009. Click here for file

Additional file 2 List of content of OSCE stations. Click here for file

Additional file 3 Follow-up of HYMS prospective cohort. Click here for file

Additional file 4 **Table S1. **Non-cognitive tests versus year 1 tutor assessment. Click here for file

Additional file 5 **Table S2. **Non-cognitive tests versus year 2 tutor assessments. Click here for file

Additional file 6 **Table S3. **Year 1 tutor assessment versus years 1 & 2 examination results. Click here for file

Additional file 7 **Table S4. **Year 2 tutor assessment (Jan and May) versus year 2 examination results. Click here for file

Additional file 8 **Table S5. **Non-cognitive tests versus year 1 & 2 examination performance. Click here for file

Additional file 9 **Table S6. **UKCAT cognitive tests versus tutor assessment. Click here for file

Additional file 10 **Table S7. **UKCAT cognitive tests versus year 1 and year 2 examination performance. Click here for file

Additional file 11 **Table S8. **Retest reliabilities of tutor ratings. Click here for file

## References

[B1] Price PB Lewis EG Loughmiller GC Nelson DE Murray MS Taylor CW Attributes of a good practicing physician J Med Educ 1971 46 229 237 5546386 10.1097/00001888-197103000-00007

[B2] Mann KV Ruedy J Millar N Andreou P Achievement of non-cognitive goals of undergraduate medical education: perceptions of medical students, residents, faculty and other health professionals Med Educ 2005 39 40 48 10.1111/j.1365-2929.2004.02031.x 15612899

[B3] General Medical Council Tomorrow’s doctors 2009 GMC, London

[B4] UK Clinical Aptitude Test http://www.ukcat.ac.uk

[B5] McManus IC Powis DA Wakeford R Ferguson E James D Richards P Intellectual aptitude tests and A levels for selecting UK school leaver entrants for medical school BMJ 2005 331 555 559 10.1136/bmj.331.7516.555 16150766 PMC1200591

[B6] Wilkinson D Zhang J Byrne GJ Medical school selection criteria and the prediction of academic performance. Evidence leading to change in policy and practice at the University of Queensland MJA 2008 188 349 354 18341459 10.5694/j.1326-5377.2008.tb01653.x

[B7] Lynch B MacKenzie R Dowell J Cleland J Prescott G Does UKCAT predict year 1 performance in medical school? Med Educ 2009 43 1203 1209 10.1111/j.1365-2923.2009.03535.x 19930512

[B8] Munro D Bore MR Powis DA Personality factors in professional ethical behaviour: studies of empathy and narcissism Aust J Psychol 2005 57 49 60 10.1080/00049530412331283453

[B9] Bore MR Munro D Kerridge I Powis DA Not moral “reasoning”: a Libertarian-Communitarian dimension of moral orientation and Schwartz’s value types Aust J Psychol 2005 57 38 48 10.1080/00049530412331283462

[B10] Powis D Bore M Munro D Lumsden MA Development of the Personal Qualities Assessment as a tool for selecting medical students J Adult and Continuing Educ 2005 11 3 14

[B11] Personal Qualities Assessment http://www.pqa.net.au

[B12] Cronbach LJ Gleser GC Psychological tests and personnel decisions 1965 University of Illinois Press, Urbana, IL

[B13] Kuncel NR Ones DS Sackett P Individual differences as predictors of work, educational, and broad life outcomes Personality and Individual Differences 2010 49 331 336 10.1016/j.paid.2010.03.042

[B14] Sherman RA Funder DC Evaluating correlations in studies of personality andbehaviour: Beyond the number of significant findings to be expected by chance J Res in Personality 2009 43 1053 1063 10.1016/j.jrp.2009.05.010

[B15] Wright SR Bradley PM Has the UK Clinical Aptitude Test improved medical student selection? Med Educ 2010 44 1069 1076 10.1111/j.1365-2923.2010.03792.x 20946477

[B16] McLachlan JC Finn G Macnaughton J The conscientiousness index: a novel tool to explore students’ professionalism Acad Med 2007 84 559 565 19704186 10.1097/ACM.0b013e31819fb7ff

[B17] Medical Schools Council Consensus statement on the role of the doctor 2008 http://www.medschools.ac.uk/AboutUs/Projects/Pages/The-Role-of-the-Doctor.aspx] accessed 22 September 2011

[B18] Norcini J The mini clinical evaluation exercise Clinical Teacher 2005 2 25 30 10.1111/j.1743-498X.2005.00060.x

