# Supplementary material for: Can personal qualities of medical students predict in-course examination success and professional behaviour? An exploratory prospective cohort study
Source: BMC Med Educ. 2012 Aug 8;12:69. doi: 10.1186/1472-6920-12-69 (PMC3473297; doi:10.1186/1472-6920-12-69)
Supplement: Additional file 1 — Examples of Tutor assessment forms. (i)Tutor assessment form, May 2008. (ii)Tutor assessment form, January 2009. (iii)Tutor assessment form, May 2009. [file 1472-6920-12-69-S1.pdf]

Additional file 1: Examples of Tutor assessment forms

(i) Tutor assessment form, May 2008

HYMS
THE HULL YORK MEDICAL SCHOOL

Summary of Personal and Professional Development

Student's Name

Tutor's Name

Please rate this student in the following areas:

		Unsatisfactory 1	Needs improvement 2	Satisfactory 3	Excellent 4
Managing self					
1	Maintains attendance	[ ]	[ ]	[ ]	[ ]
2	Attends punctually	[ ]	[ ]	[ ]	[ ]
3	Dresses appropriately for all activities	[ ]	[ ]	[ ]	[ ]
4	Treats others with appropriate respect	[ ]	[ ]	[ ]	[ ]
5	Demonstrates appropriate attitudes	[ ]	[ ]	[ ]	[ ]
6	Completes given tasks on time	[ ]	[ ]	[ ]	[ ]
7	Acknowledges weaknesses and attempts to improve	[ ]	[ ]	[ ]	[ ]
Group / teamwork					
8	Integrates themselves into the group	[ ]	[ ]	[ ]	[ ]
9	Takes responsibility for group learning	[ ]	[ ]	[ ]	[ ]
10	Contributes work for the group	[ ]	[ ]	[ ]	[ ]
11	Treats peers with respect	[ ]	[ ]	[ ]	[ ]
12	Listens effectively	[ ]	[ ]	[ ]	[ ]
13	Contributes to a positive learning atmosphere	[ ]	[ ]	[ ]	[ ]
14	Willing to learn from others	[ ]	[ ]	[ ]	[ ]
15	Communicates appropriately with peers	[ ]	[ ]	[ ]	[ ]
16	Communicates appropriately with tutors and other HYMS staff	[ ]	[ ]	[ ]	[ ]
17	Manages conflict appropriately	[ ]	[ ]	[ ]	[ ]

In my opinion, this student is (pick one)

A particularly promising student [ ]

An average student [ ]

A problematic student [ ]

(ii) Tutor assessment form, January 2009

HYMS
THE HULL YORK MEDICAL SCHOOL

Tutor Assessment of Student Professional Behaviours

Student name:

Tutor name:

Instance:

This student was rated as follows:	Below expectation 1	2	Borderline 3	Meets expectations 4	Above expectation 5
**Managing self**					
1 Attends punctually	☐	☐	☐	☐	☐
2 Treats tutors with appropriate respect	☐	☐	☐	☐	☐
3 Demonstrates appropriate attitudes	☐	☐	☐	☐	☐
4 Completes given tasks on time	☐	☐	☐	☐	☐
**Group / teamwork**					
5 Integrates themselves into the group	☐	☐	☐	☐	☐
6 Takes responsibility for group learning	☐	☐	☐	☐	☐
7 Contributes work for the group	☐	☐	☐	☐	☐
8 Treats peers with respect	☐	☐	☐	☐	☐
9 Listens effectively	☐	☐	☐	☐	☐
10 Willing to learn from others	☐	☐	☐	☐	☐
11 Undertakes PBL roles appropriately	☐	☐	☐	☐	☐
12 Communicates appropriately with peers	☐	☐	☐	☐	☐
13 Communicates appropriately with tutors	☐	☐	☐	☐	☐
14 Manages conflict appropriately	☐	☐	☐	☐	☐

Tutor comments:

Student comments:

Adapted from Peer Assessment of Professional Behaviours Form created by Gary Butler, University of Wollongong, used with permission

(iii) **Tutor assessment form, May 2009**

H·Y·M·S
THE HULL YORK MEDICAL SCHOOL

Tutor Assessment of Student Professional Behaviours

Student name:

Tutor name:

Instance:

**ROA forms**			
**Form**	**Present**	**Complete**	**If incomplete then brief details (major or minor deficiency):**
Form A:	☐	☐	
Form B:	☐	☐	
Form C:	☐	☐	

	This student was rated as follows:	Unsatisfactory<br>**1**	Borderline<br>**2**	Satisfactory<br>**3**	Excellent<br>**4**
	**Managing self**				
1	Maintains attendance	☐	☐	☐	☐
2	Attends punctually	☐	☐	☐	☐
3	Dresses appropriately for all activities	☐	☐	☐	☐
4	Treats others with appropriate attitudes	☐	☐	☐	☐
5	Demonstrates appropriate attitudes	☐	☐	☐	☐
6	Completes given tasks and hands in paperwork on time	☐	☐	☐	☐
7	Acknowledges weaknesses/takes feedback on board to improve	☐	☐	☐	☐
	**Group / teamwork**				
8	Integrates themselves into the group	☐	☐	☐	☐
9	Takes responsibility for group learning	☐	☐	☐	☐
10	Contributes work for the group	☐	☐	☐	☐
11	Treats peers with respect	☐	☐	☐	☐
12	Listens effectively	☐	☐	☐	☐
13	Contributes to a positive learning atmosphere	☐	☐	☐	☐
14	Is willing to learn from others	☐	☐	☐	☐
15	Communicates appropriately with peers	☐	☐	☐	☐
16	Communicates appropriately with tutors	☐	☐	☐	☐
17	Manages conflict appropriately	☐	☐	☐	☐
	**Clinical / patient**				
18	Undertakes clinical skills practice conscientiously	☐	☐	☐	☐
19	Engages in clinical placements appropriately	☐	☐	☐	☐
20	Demonstrates appropriate professional behavious in clinical encounters (eg: with SP's, patients, HVs, etc)	☐	☐	☐	☐

Additional confidential question: In my opinion this student is ... (pick one)

A particularly promising student ☐
An average student ☐
A problematic student ☐
