# Supplementary material for: Can personal qualities of medical students predict in-course examination success and professional behaviour? An exploratory prospective cohort study
Source: BMC Med Educ. 2012 Aug 8;12:69. doi: 10.1186/1472-6920-12-69 (PMC3473297; doi:10.1186/1472-6920-12-69)
Supplement: Additional file 2 — List of content of OSCE stations. [file 1472-6920-12-69-S2.pdf]

Additional file 2: List of content of OSCE stations.

**OSCE Stations, Year 1**	**OSCE Stations, Year 2**
**Communication Skills**<br>Exploring current problem<br>Exploring family history<br>Using empathy<br>Exploring patient’s lifestyle<br>Interviewing a depressed patient<br>Exploring chronic illness<br>Exploring sensitive topic	**Communication Skills**<br>Explaining chest problem<br>Exploring place of birth<br>Providing heart problem information<br>Explaining heart problem<br>Explaining pain symptoms<br>Explaining consequences of alcohol use
**Practical Skills**<br>Chest examination<br>Measuring blood pressure<br>Basic life support<br>Neurological examination<br>Examination of spine<br>Measuring expiratory peak flow<br>Examination of the abdomen	**Practical Skills**<br>Basic life support<br>Cranial nerve examination<br>Neurological examination<br>General physical examination<br>Examination of respiratory system<br>Examination of the abdomen
