# Supplementary material for: Can personal qualities of medical students predict in-course examination success and professional behaviour? An exploratory prospective cohort study
Source: BMC Med Educ. 2012 Aug 8;12:69. doi: 10.1186/1472-6920-12-69 (PMC3473297; doi:10.1186/1472-6920-12-69)

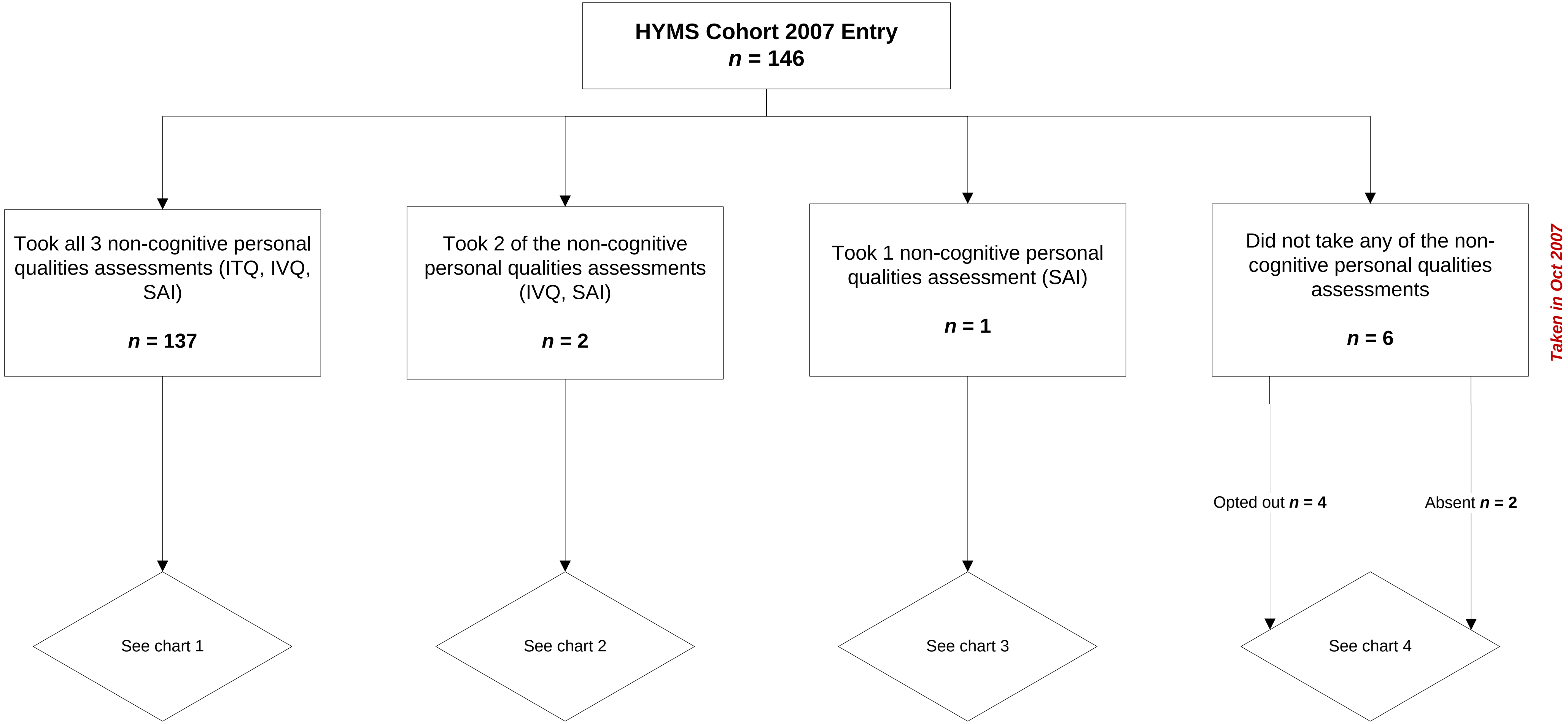
HYMS Cohort 2007 Entry
n = 146
Took all 3 non-cognitive personal qualities assessments (ITQ, IVQ, SAI)
n = 137
Took 2 of the non-cognitive personal qualities assessments (IVQ, SAI)
n = 2
Took 1 non-cognitive personal qualities assessment (SAI)
n = 1
Did not take any of the non-cognitive personal qualities assessments
n = 6
Taken in Oct 2007
Opted out n = 4
Absent n = 2
See chart 1
See chart 2
See chart 3
See chart 4

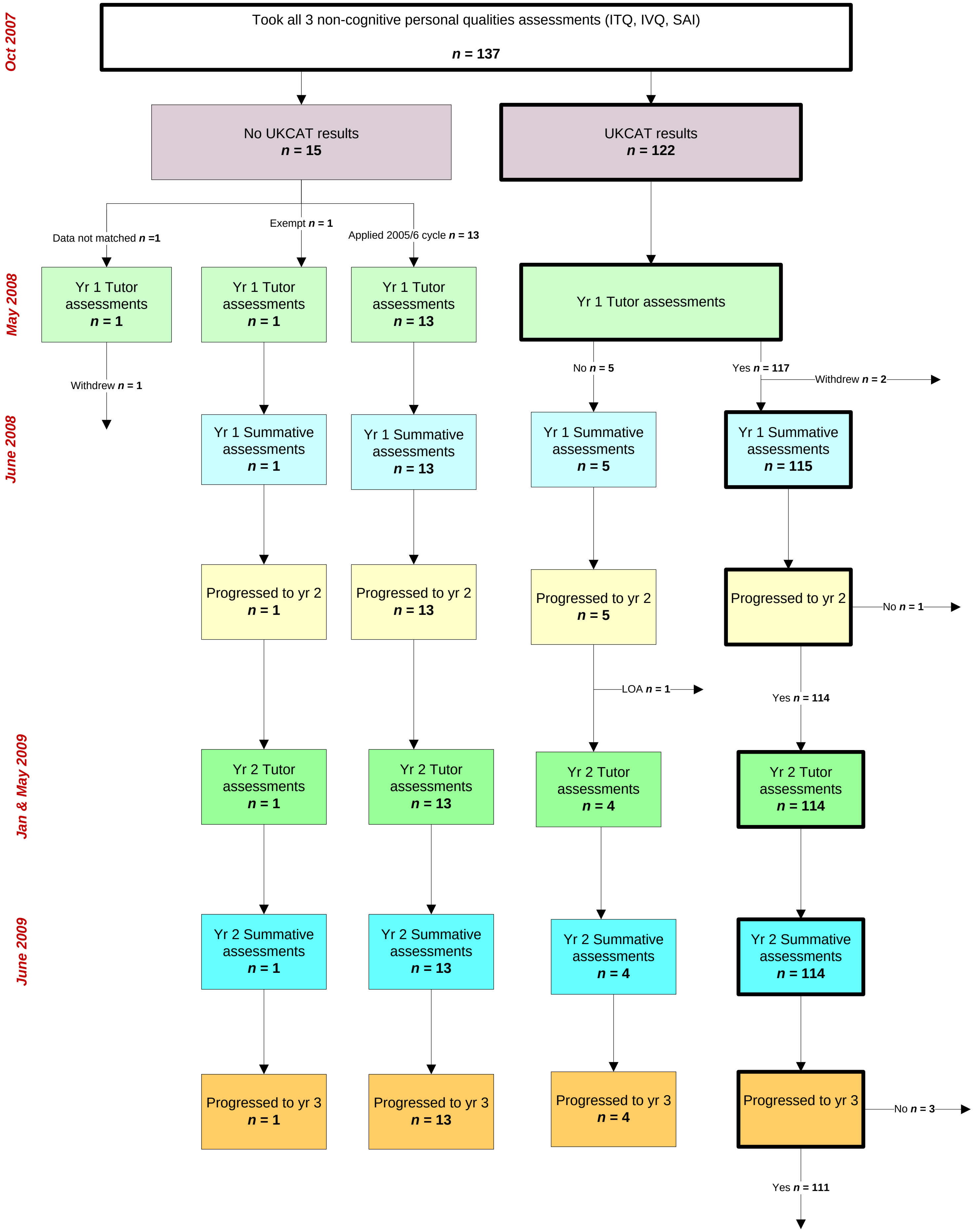
Oct 2007
Took all 3 non-cognitive personal qualities assessments (ITQ, IVQ, SAI)
n = 137
No UKCAT results
n = 15
UKCAT results
n = 122
Data not matched n =1
Exempt n = 1
Applied 2005/6 cycle n = 13
May 2008
Yr 1 Tutor assessments n = 1
Yr 1 Tutor assessments n = 1
Yr 1 Tutor assessments n = 13
Yr 1 Tutor assessments
Withdrew n = 1
No n = 5
Yes n = 117
Withdrew n = 2
June 2008
Yr 1 Summative assessments n = 1
Yr 1 Summative assessments n = 13
Yr 1 Summative assessments n = 5
Yr 1 Summative assessments n = 115
Progressed to yr 2 n = 1
Progressed to yr 2 n = 13
Progressed to yr 2 n = 5
Progressed to yr 2
No n = 1
LOA n = 1
Yes n = 114
Jan & May 2009
Yr 2 Tutor assessments n = 1
Yr 2 Tutor assessments n = 13
Yr 2 Tutor assessments n = 4
Yr 2 Tutor assessments n = 114
June 2009
Yr 2 Summative assessments n = 1
Yr 2 Summative assessments n = 13
Yr 2 Summative assessments n = 4
Yr 2 Summative assessments n = 114
Progressed to yr 3 n = 1
Progressed to yr 3 n = 13
Progressed to yr 3 n = 4
Progressed to yr 3
No n = 3
Yes n = 111

HYMS Prospective Cohort Study Chart 2

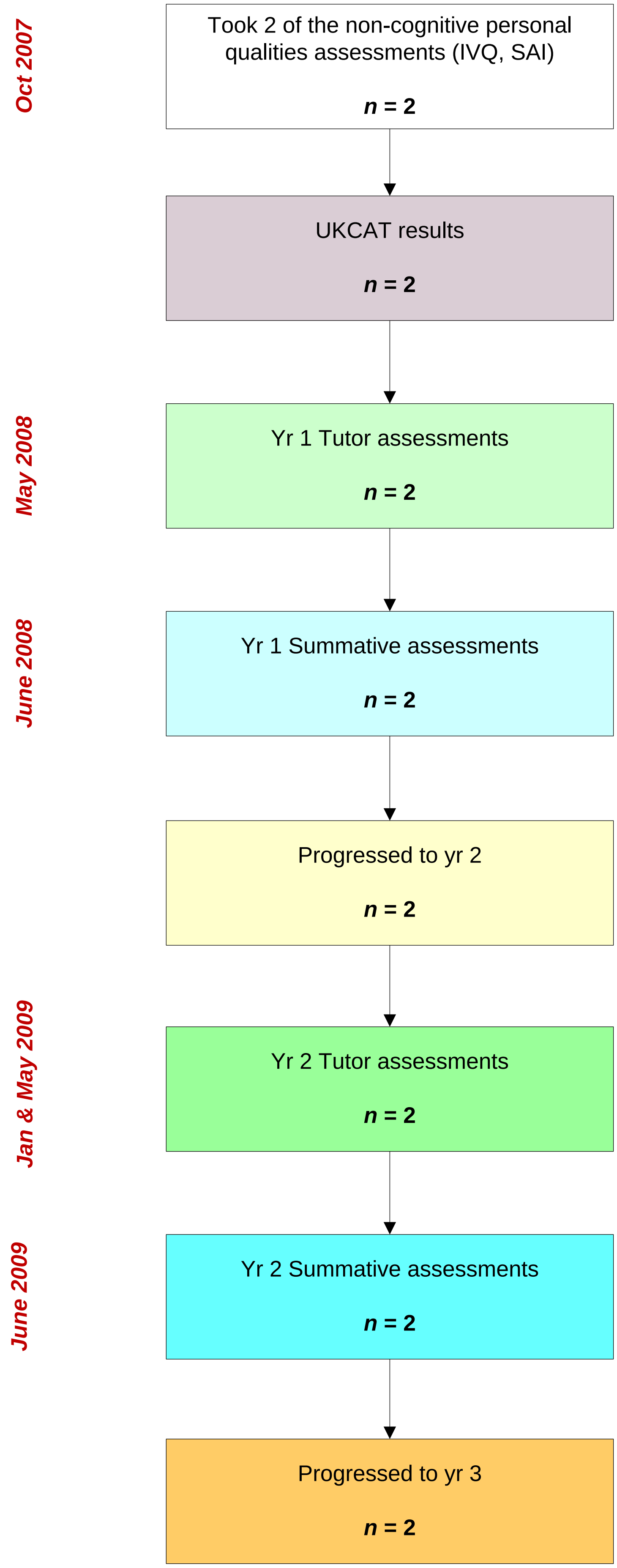

HYMS Prospective Cohort Study Chart 3

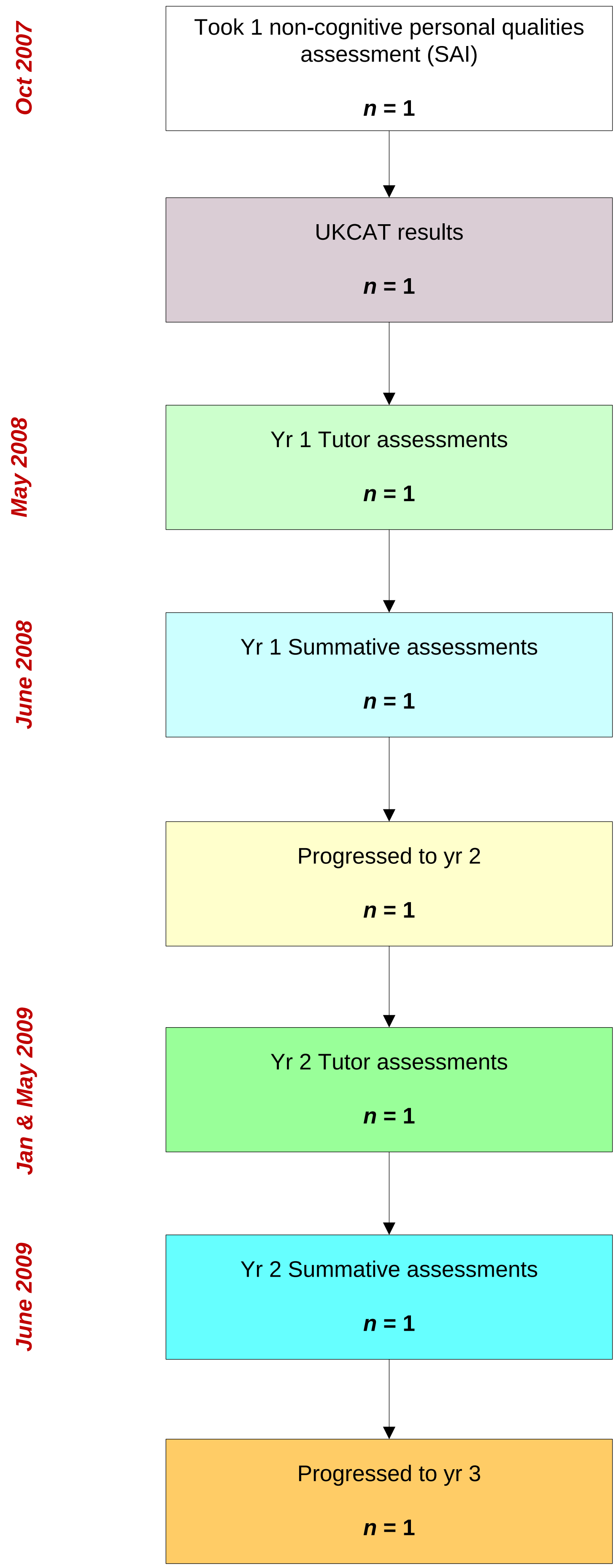

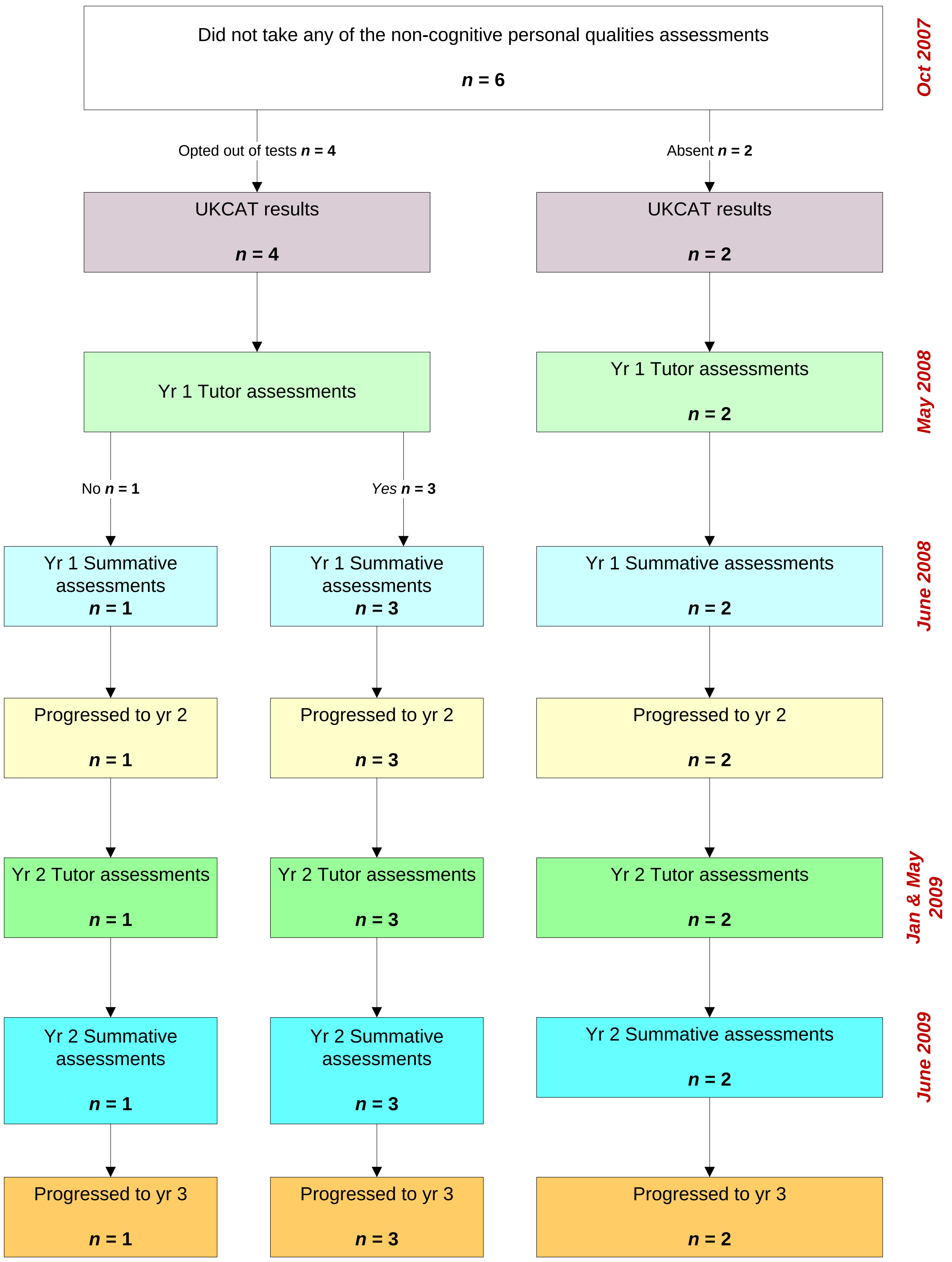
Did not take any of the non-cognitive personal qualities assessments
n = 6
Oct 2007
Opted out of tests n = 4
Absent n = 2
UKCAT results
n = 4
UKCAT results
n = 2
Yr 1 Tutor assessments
Yr 1 Tutor assessments
n = 2
May 2008
No n = 1
Yes n = 3
Yr 1 Summative assessments
n = 1
Yr 1 Summative assessments
n = 3
Yr 1 Summative assessments
n = 2
June 2008
Progressed to yr 2
n = 1
Progressed to yr 2
n = 3
Progressed to yr 2
n = 2
Yr 2 Tutor assessments
n = 1
Yr 2 Tutor assessments
n = 3
Yr 2 Tutor assessments
n = 2
Jan & May 2009
Yr 2 Summative assessments
n = 1
Yr 2 Summative assessments
n = 3
Yr 2 Summative assessments
n = 2
June 2009
Progressed to yr 3
n = 1
Progressed to yr 3
n = 3
Progressed to yr 3
n = 2

Numbers contributing to each outcome measure

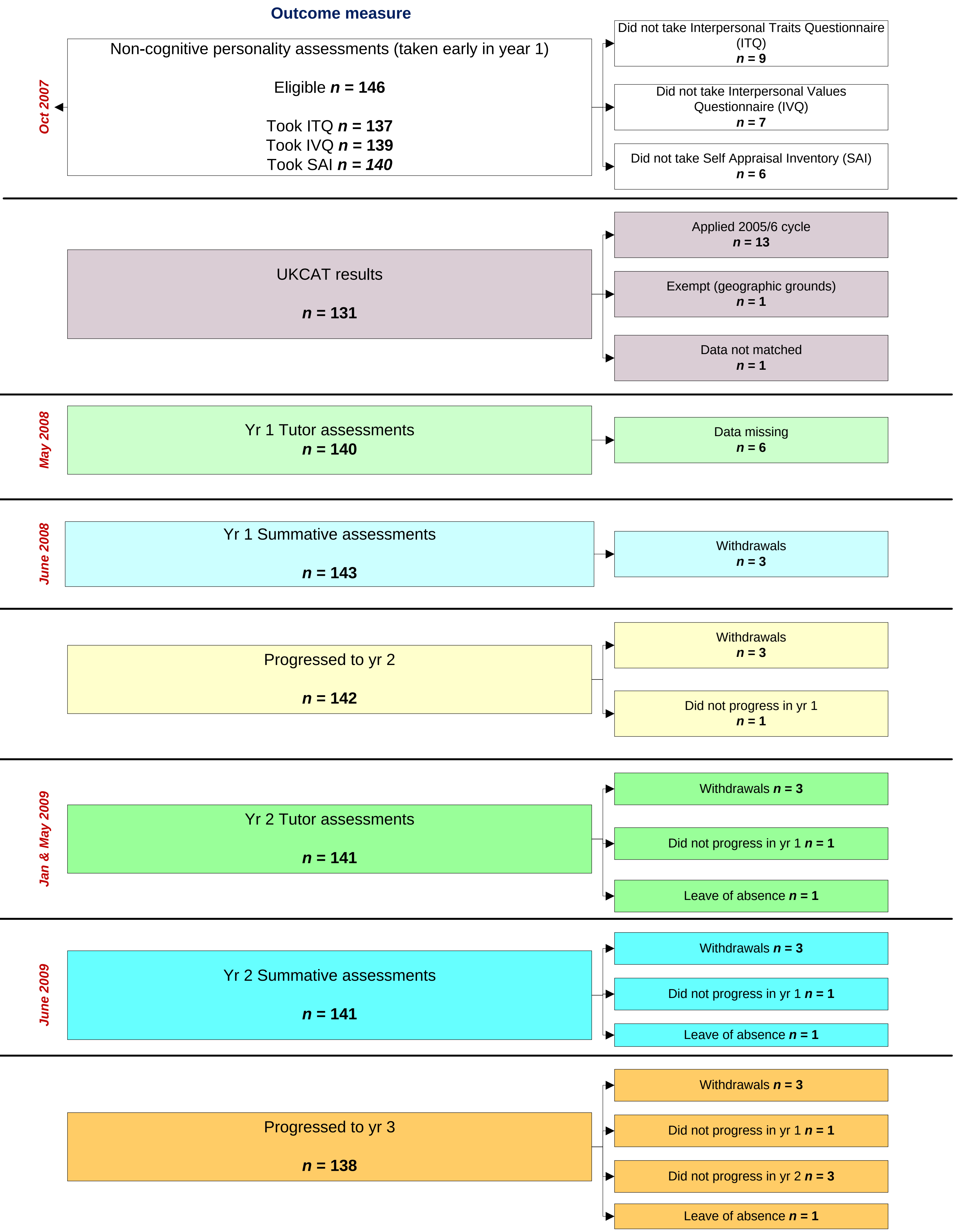

Supplement: Additional file 3 — Follow-up of HYMS prospective cohort. [file 1472-6920-12-69-S3.pdf]
