# Supplementary material for: Can personal qualities of medical students predict in-course examination success and professional behaviour? An exploratory prospective cohort study
Source: BMC Med Educ. 2012 Aug 8;12:69. doi: 10.1186/1472-6920-12-69 (PMC3473297; doi:10.1186/1472-6920-12-69)
Supplement: Additional file 4 — Table S1. Non-cognitive tests versus year 1 tutor assessment. [file 1472-6920-12-69-S4.pdf]

Table S1 Non-cognitive tests versus year 1 tutor assessment

Tutor assessment item	1	2	3	4	5	6	7	8	Overall
**Non-cognitive test Scales from SAI**									
Conscientiousness		-.179*							
Irrational thinking	-.305***	-.237**		-.198*	-.245***	-.224**	-.179*		
Neuroticism		-.219*			-.200*			-.175*	
Moodiness		-.182*						-.184*	
RESILIENCE		+.216*			+.205*			+.186*	
**Non-cognitive test Scales from ITQ**									
Narcissism			-.231**						
Aloofness									
Confidence				-.186*					
Empathy									
INVOLVEMENT									+.177*

N = 134 * p < .05; ** p < .01; *** p < .001

Key to year 1 tutor assessment items

1 Demonstrates appropriate attitudes
2 Integrates into group
3 Acknowledges weaknesses and accepts feedback
4 Treats peers with respect
5 Listens effectively
6 Communicates appropriately with peers
7 Communicates appropriately with tutors
8 Manages conflict appropriately
Overall tutor rating May 2008

Note

6 of 11 SAI scales correlated with no tutor assessment items.
The IVQ scale correlated with no tutor assessment items.
8 of 17 tutor assessment items correlated with no non-cognitive test scale.
