# Supplementary material for: Can personal qualities of medical students predict in-course examination success and professional behaviour? An exploratory prospective cohort study
Source: BMC Med Educ. 2012 Aug 8;12:69. doi: 10.1186/1472-6920-12-69 (PMC3473297; doi:10.1186/1472-6920-12-69)
Supplement: Additional file 5 — Table S2. Non-cognitive tests versus year 2 tutor assessments. [file 1472-6920-12-69-S5.pdf]

Table S2 Non-cognitive tests versus year 2 tutor assessments: *Jan 09 in top row of cell*, *italicised*; May 09 in lower row.

Tutor assessment item (key below)	1	2	3	4	5	6	7	8	9	10	11	Overall Jan'09	Overall May '09
**Non-cognitive tests**													
**Scales from SAI**													
Conscientiousness	.												
	.21 *	.18*		.23**	.21*								
Permissiveness					*.24****			*.21**					
					.24**								
Anti-social behaviour										*.21**			
				.21*									
Restraint				*.26****								***.20****	
	.20*	.21*	.23**	.24**	.35***						.18*		**.20***
SELF-CONTROL				*.20**				*.19**					
**Scales from ITQ**													
Narcissism										*.17**			
Aloofness										*.22**			
Confidence						*.17**	*.19**	*.26****					
						.18*			.20*				**.31*****
Empathy										*.18**			
INVOLVEMENT										*.17**			
**Scales from IVQ**													
Social responsibility													
	.21*	.19*	.24**										

N = 134 Correlations are rounded to 2 significant places and negative correlations are shown shaded.
* p < .05; ** p < .01; *** p < .001

Note 5 of 10 SAI scales correlated with no tutor assessment items
7 of 14 (Jan 09) and 9 of 17 (May 09) tutor assessment items correlated with no non-cognitive test scale

Key to year 2 tutor assessment items

1 Maintains attendance
2 Attends punctually
3 Dresses appropriately for all activities
4 Completes tasks and hands in work on time
5 Acknowledges weaknesses and accepts feedback
6 Contributes work for group
7 Treats peers with respect
8 Listens effectively
9 Contributes to positive learning atmosphere
10 Manages conflict appropriately
11 Engages in clinical placements appropriately
