# Supplementary material for: Can personal qualities of medical students predict in-course examination success and professional behaviour? An exploratory prospective cohort study
Source: BMC Med Educ. 2012 Aug 8;12:69. doi: 10.1186/1472-6920-12-69 (PMC3473297; doi:10.1186/1472-6920-12-69)
Supplement: Additional file 6 — Table S3. Year 1 tutor assessment versus years 1 & 2 examination results. [file 1472-6920-12-69-S6.pdf]

Table S3 Year 1 tutor assessment versus years 1 & 2 examination results

Year 1 exam data in _top row of each cell, italicised;_ year 2 exam data in lower row

**Examination results**	Theme A score	OSCE Com skills	OSCE Prac skills	Theme B score	Theme C score	Overall Exam score	Top 20% versus Bottom 20%†
**Tutor assessment item**							
Attends punctually							
			+.207*				
Appropriate attitudes							
	+.192*					+.189*	
Integrates into group	_+.324***_	_+.228**_	_+.179*_	_+.295***_		_+.338***_	_10.43**_
	+.288***		+.176*	+.248***	+.234**	+.312***	6.45*
Takes responsibility	_+.280***_	_+.195*_	_+.244**_	_+.230**_		_+.274***_	_6.61*_
for group learning	+.173*		+.284***			+.188*	4.68*
Contributes work	_+.365***_	_+.271***_	_+.234**_	_+.335***_		_+.377***_	_17.11***_
for group	+.267***		+.297***	+.176*	+.231**	+.280***	12.21***
Contributes to positive	_+.338***_	_+.198*_			_+.213*_	_+.322***_	_10.75**_
learning atmosphere	+.339***		+.216*	+.234**	+.247***	+.345***	12.00***
Acknowledges own	_+.252***_				_+.185*_	_+.258***_	_10.25**_
weaknesses	+.246***			+.177*	+.185*	+.254***	9.08**
Listens effectively	_+.228**_					_+.195*_	_5.93*_
	+.289***				+.182*	+.262***	5.58*
Willing to learn	_+.208*_					_+.193*_	_5.81*_
from others	+.268***				+.224**	+.253***	
Communicates appro-	_+.197*_					_+.173*_	
-priately with peers							
Manages conflict	_+.226**_					_+.222**_	
appropriately				+.216*		+.197*	
Overall tutor rating	_+.363***_			_+.215*_		_+.362***_	_23.21***_
May 2008	+.268***			+.169*		+.239**	7.09**

N = 135 - 137 * p < .05; ** p < .01; *** p < .001; † F value

Note 6 of 17 tutor assessment items correlated with no examination score
All examination scores correlated with some tutor assessment items
