# Supplementary material for: Can personal qualities of medical students predict in-course examination success and professional behaviour? An exploratory prospective cohort study
Source: BMC Med Educ. 2012 Aug 8;12:69. doi: 10.1186/1472-6920-12-69 (PMC3473297; doi:10.1186/1472-6920-12-69)
Supplement: Additional file 7 — Table S4. Year 2 tutor assessment (Jan and May) versus year 2 examination results. [file 1472-6920-12-69-S7.pdf]

Table S4 Year 2 tutor assessment (Jan and May) versus year 2 examination results

Jan 09 data in top row of each cell, italicised; May 09 data in lower row

**Examination results**	Theme A score	OSCE Com skills	OSCE Prac skills	Theme B score	Theme C score	Overall Exam score	Top 20% versus Bottom 20%†
**Tutor assessment item**							
Attends punctually		*+.250****	*+.217**		*+.232***		
Dresses appropriately				*-.166**			
for all activities			-.186*	-.204*			
Appropriate attitudes			*+.195**				
							4.98*
Integrates into group							
			+.188*				
Takes responsibility		*+.224***	*+.196**	*+.222***		*+.197**	*5.07**
for group learning							
Contributes work	*+.235***	*+.210**	*+.341****	*+.224***		*+.246****	
for group	+.168*		+.183*			+.172*	5.74*
Contributes to positive							
learning atmosphere	+.187*				+.177*	+.177*	
Acknowledges own							
weaknesses			-.205*				
Listens effectively							
				-.198*		-.189*	
Willing to learn							
from others			-.222**	-.286***		-.181*	
Manages conflict	*+.215**	*+.217***		*+.284****	*+.261****	*+.280****	5.44*
appropriately							
Overall tutor rating		*+.170**	*+.299****				
May 2008	+.177*					+.174*	

N = 135 - 137   * p < .05; ** p < .01; *** p < .001; † F value

Note 9 of 14 (Jan 2009) tutor assessment items correlated with no examination score
10 of 17 (May 2009) tutor assessment items correlated with no examination score
All examination scores correlated with some tutor assessment items
