# Supplementary material for: Can personal qualities of medical students predict in-course examination success and professional behaviour? An exploratory prospective cohort study
Source: BMC Med Educ. 2012 Aug 8;12:69. doi: 10.1186/1472-6920-12-69 (PMC3473297; doi:10.1186/1472-6920-12-69)
Supplement: Additional file 8 — Table S5. Non-cognitive tests versus year 1 & 2 examination performance. [file 1472-6920-12-69-S8.pdf]

Table S5 Non-cognitive tests versus year 1 & 2 examination performance

Year 1 exam data in *top row of each cell, italicised;* year 2 exam data in lower row

**Examination results**	Theme A scores	OSCE com skills	OSCE prac skills	Theme B scores	Theme C scores	Overall exam score	Top 20% versus bottom 20%
**Non-cognitive test Scales from SAI**							
Conscientiousness							
			+.187*				
Irrational thinking		*-.171**					
	-.174*	-.201*		-.176*		-.186*	4.92*
Permissiveness		*-.203**					
Lie scale				*-.216**			
	+.226*						
**Non-cognitive test Scales from ITQ**							
Narcissism							*6.66**
		-.209*		-.201*		-.188*	
Aloofness	*-.183**			*-.228***		*-.202**	*11.21****
	-.192*	-.201*		-.173*		-.180*	
Confidence			*+.274****				
Empathy							
INVOLVEMENT			*+.206**	*+.194+*			*8.52***

N = 134 – 137 * p < .05; ** p < .01; *** p < .001; † F value

Note 7 of 11 SAI scales correlated with no examination score
The IVQ scale correlated with no examination score
All examination scores, except Theme C, correlated with some non-cognitive test scale
