# Supplementary material for: Can personal qualities of medical students predict in-course examination success and professional behaviour? An exploratory prospective cohort study
Source: BMC Med Educ. 2012 Aug 8;12:69. doi: 10.1186/1472-6920-12-69 (PMC3473297; doi:10.1186/1472-6920-12-69)
Supplement: Additional file 9 — Table S6. UKCAT cognitive tests versus tutor assessment. [file 1472-6920-12-69-S9.pdf]

Table S6 UKCAT cognitive tests versus tutor assessment

Year 2
Jan ’09 italicised in top row of cell, May ’09 in lower row

**Tutor assessment items**	**Year 1**	**Year 2**						
		1	2	3	4	5	6	7
**UKCAT cognitive tests**								
Decision analysis					+.197*			_+.219*_
Quantitative reasoning							-.190*	
Abstract reasoning		+.177*	+.247**	+.177*		+.241**		
Verbal reasoning	-.218*							
Overall cognitive ability					+.187*			

N = 125 – 128 * p < .05; ** p < .01

Note
16 of 17 May 2008 TA items correlated with no UKCAT cognitive test scale
13 of 14 Jan 2009 TA items correlated with no UKCAT cognitive test scales
11 of 17 May 2009 TA items correlated with no UKCAT cognitive test scales

Key to year 1 tutor assessment items
Treats tutors with respect

Key to year 2 tutor assessment items
1 Demonstrates appropriate attitudes
2 Integrates into group
3 Takes responsibility for group learning
4 Contributes work for group
5 Contributes to positive learning atmosphere
6 Willing to learn from others
7 Manages conflict appropriately
