# Supplementary material for: Can personal qualities of medical students predict in-course examination success and professional behaviour? An exploratory prospective cohort study
Source: BMC Med Educ. 2012 Aug 8;12:69. doi: 10.1186/1472-6920-12-69 (PMC3473297; doi:10.1186/1472-6920-12-69)
Supplement: Additional file 10 — Table S7. UKCAT cognitive tests versus year 1 and year 2 examination performance. [file 1472-6920-12-69-S10.pdf]

Table S7 UKCAT cognitive tests versus year 1 and year 2 examination performance

Year 1 italicised in top row of cell, year 2 in lower row of cell

**Examination results**	Theme A score	OSCE Com skills	OSCE Prac skills	Theme B score	Theme C score	Overall Exam score	Top 20% versus Bottom 20%†
**UKCAT cognitive tests**							
Decision analysis	*+.181**		*+.197**		*+.180**	*+.209**	*7.36***
	+.258**			+.281***	+.204*	+.291***	12.65***
Quantitative reasoning					*+.233***		
				+.275**	+.267**	+.200*	11.70***
Abstract reasoning	*+.234***				*+.271***	*+.255***	
					+.202*		
Verbal reasoning					*+.363****		
				+.331***	+.253**	+.241**	5.61*
Overall cognitive ability	*+.214**				*+.390****	*+.277****	*9.24***
	+.212*			+.377***	+.343***	+.323***	18.46***

N = 129 - 131 * p < .05; ** p < .01; *** p < .001; † F value
