# Supplementary material for: Can personal qualities of medical students predict in-course examination success and professional behaviour? An exploratory prospective cohort study
Source: BMC Med Educ. 2012 Aug 8;12:69. doi: 10.1186/1472-6920-12-69 (PMC3473297; doi:10.1186/1472-6920-12-69)
Supplement: Additional file 11 — Table S8. Retest reliabilities of tutor ratings. [file 1472-6920-12-69-S11.pdf]

Table S8 Retest reliabilities of tutor ratings

Tutor assessment item with same (or similar) wording	May 08 versus Jan 09	May 08 versus May 09	Jan 09 versus May 09
Attends punctually	+.372***	+.208 *	+.441***
Demonstrates appropriate attitudes	+.239 **	N.A.	+.296***
Integrates into group	+.304***	+.290***	+.351***
Takes responsibility for group learning	+.269 **	+.082	+.378***
Contributes work for group	+.378***	+.344***	+.400***
Treats peers with respect	+.123	+.114	+.152
Listens effectively	+.123	+.083	+.190 *
Willing to learn from others	+.089	+.064	+.281***
Communicates appropriately with peers	+.011	+.181 *	+.192 *
Communicates appropriately with tutors	–.059	–.003	+.261 **
Contributes to a positive learning atmosphere	N.A.	+.178 *	N.A.
Manages conflict appropriately	+.169 *	+.174 *	+.015
Treats tutors with appropriate respect / communicates appropriately with tutors	N.A.	N.A.	+.492***
Acknowledges weakness and takes feedback on board	N.A.	+.096	N.A.
**Overall Tutor Rating**	**+.310*** **	**+.363*** **	**+.674*** **

N = 136-141 Pearson product-moment correlation coefficient, 2-tailed;
* p < .05; ** p < .01; *** p < .001; N.A. = measure not used on both occasions
